# Probiotic supplementation does not improve eradication rate of *Helicobacter pylori* infection compared to placebo based on standard therapy: a meta-analysis

**DOI:** 10.1038/srep23522

**Published:** 2016-03-21

**Authors:** Chao Lu, Jianzhong Sang, Haijian He, Xingyong Wan, Yiming Lin, Lan Li, Youming Li, Chaohui Yu

**Affiliations:** 1 Department of Gastroenterology, the First Affiliated Hospital, College of Medicine, Zhejiang University, Hangzhou, China

## Abstract

This meta-analysis included eligible randomized controlled trials (RCTs) with the aim of determining whether probiotic supplementation can improve *H. pylori* eradication rates. PUBMED, EBSCO, Web of Science, and Ovid databases were searched. We included RCTs that investigated the effect of combining probiotics, with or without a placebo, with standard therapy. A total of 21 RCTs that reported standard therapy plus probiotics were included. Compared to the placebo group, the probiotics group was 1.21(OR 1.21, 95% CI: 0.86, 1.69) and 1.28 (OR 1.28, 95% CI: 0.88, 1.86) times more likely to achieve eradication of *H. pylori* infection in intent-to-treat (ITT) analysis and per protocol (PP) analysis, respectively. Probiotics with triple therapy plus a 14-day course of treatment did not improve the eradication of *H. pylori* infection (OR 1.44, 95% CI: 0.87, 2.39) compared to the placebo. Moreover, the placebo plus standard therapy did not improve eradication rates compared to standard therapy alone (*P* = 0.816). However, probiotics did improve the adverse effects of diarrhea and nausea. These pooled data suggest that the use of probiotics plus standard therapy does not improve the eradication rate of *H. pylori* infection compared to the placebo.

*Helicobacter pylori* (*H. pylori*) is a Gram-negative microaerophilic bacterium that dwells in the human gastric mucosa. It is commonly associated with gastroduodenal diseases in humans such as gastric mucosa-associated lymphoid tissue lymphoma^1^, peptic ulcer disease^2^, and even gastric cancer^3^,^4^. Almost 50% of the worldwide human population is infected, with people living in developing countries showing higher rates of infection^5^. Triple therapy, which has been proposed as a first approach for *H. pylori* eradication, includes a proton pump inhibitor (PPI), clarithromycin and either amoxicillin or metronidazole. Other choices include sequential therapy and quadruple therapy^6^. However, the eradication rate using standard therapy was reported to be unsatisfactory using first-line or second-line treatments due to increased resistance to antibiotics and patient non-compliance^7^,^8^,^9^. Probiotics appear to be promising supplements for standard therapy of *H. pylori* infection.

Probiotics are defined as living microbial species that can induce anti-inflammatory and anti-oxidative mechanisms that may improve bowel microecology and general health^10^,^11^. Probiotics contain Lactobacillus, Saccharomyces boulardii, Bifidobacterium, and other bacteria and yeasts. Some meta-analyses have reported that probiotic supplementation can improve the eradication rate of *H. pylori* compared to standard therapy alone^12^,^13^,^14^. It is widely accepted that probiotics can improve *H. pylori* eradication and reduce side effects during standard therapy.

However, we found that the control groups in RCTs in previous meta-analyses were mostly without a placebo. Placebo preparations matched the probiotic preparation in color, size, shape and weight, and had no pharmacological effect. Surprisingly, we found that the eradication rate of *H. pylori* had no statistical significance between probiotic supplementation groups and placebo supplementation groups in most studies. A placebo may also influence the eradication rate of *H. pylori* by a placebo effect acting through the alteration of systemic and enteric levels of hormones^15^. Nevertheless, there is no direct research on placebo and *H.pylori* to support this viewpoint.

This study aimed to select RCTs, and establish whether probiotic supplementation could improve tolerance to *H. pylori* standard triple eradication therapy compared to the placebo. We included RCTs without a placebo for comparison.

## Results

### Study characteristics

**Figure 1:**
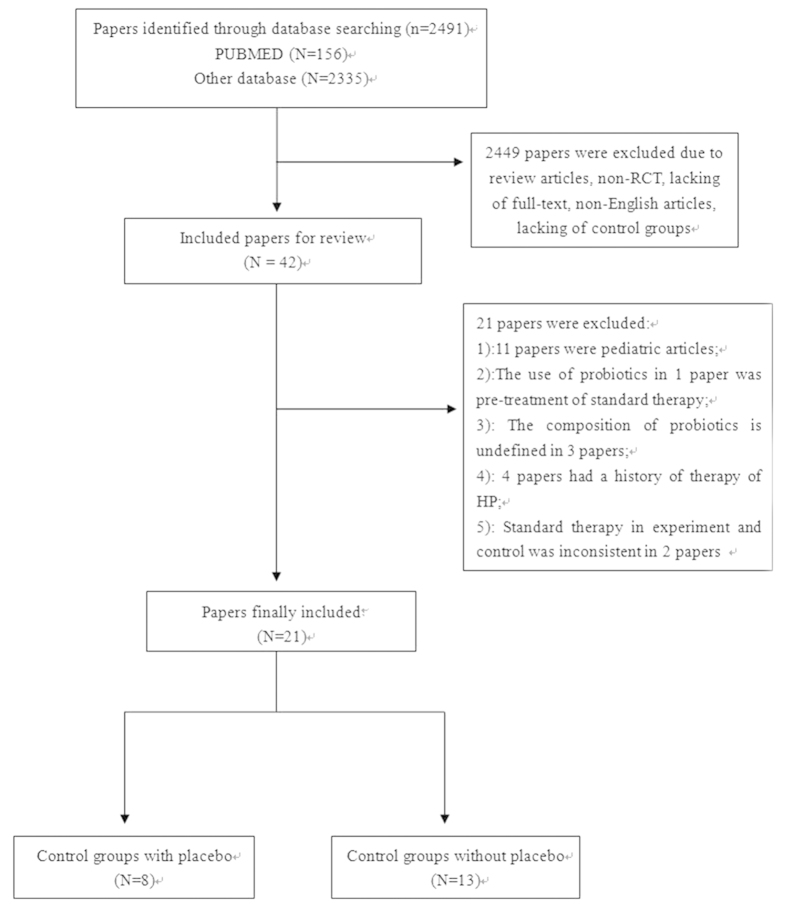
Flow diagram for searching studies.

Our search identified 2,491 references, of which eight studies with placebo groups^16^,^17^,^18^,^19^,^20^,^21^,^22^,^23^ and 13 studies without placebo groups^24^,^25^,^26^,^27^,^28^,^29^,^30^,^31^,^32^,^33^,^34^,^35^,^36^ met our inclusion criteria (Fig. 1). Reasons for exclusion are shown in Fig. 1. Study characteristics of therapeutic regimens are shown in Tables 1 and 2. Geographically, studies mainly originated from Europe (13/21), with other studies originating from South America (1/21) and Asia (7/21). Twenty studies used standard triple therapy and one used bismuth-quadruple therapy. ^13^C-urea breath test (^13^C-UBT) was the main diagnostic method selected. A total of 3,520 participants were included in our research, in which 3,349 participants completed their respective trial. The terminal point of follow-up was reexamination of *H. pylori* infection after standard therapy, which ranged from 4 weeks to 10 weeks after the end of treatment. Characteristics of age, gender and type of patients are shown in Table 3. We found no significant difference in age (SMD = −0.05, 95% CI = −0.19, 0.09) or gender (OR = 0.92, 95% CI: 0.8, 1.06) between the two groups. Ten studies used a single probiotic and 11 used compound probiotics. Placebos were administered in the same number of sachets as the probiotics. Boxes containing active study treatments, and placebos were identical in color, size, shape, weight and taste, and contained the same number of sachets. No trademark identifications were present, either on the probiotic or the placebo sachets. The composition of a placebo in one study was capsules of acidified milk powder (skim milk biologically acidified by commercial yogurt culture)^21^, of which no therapeutic effect was mentioned.

### Eradication Rates

**Figure 2:**
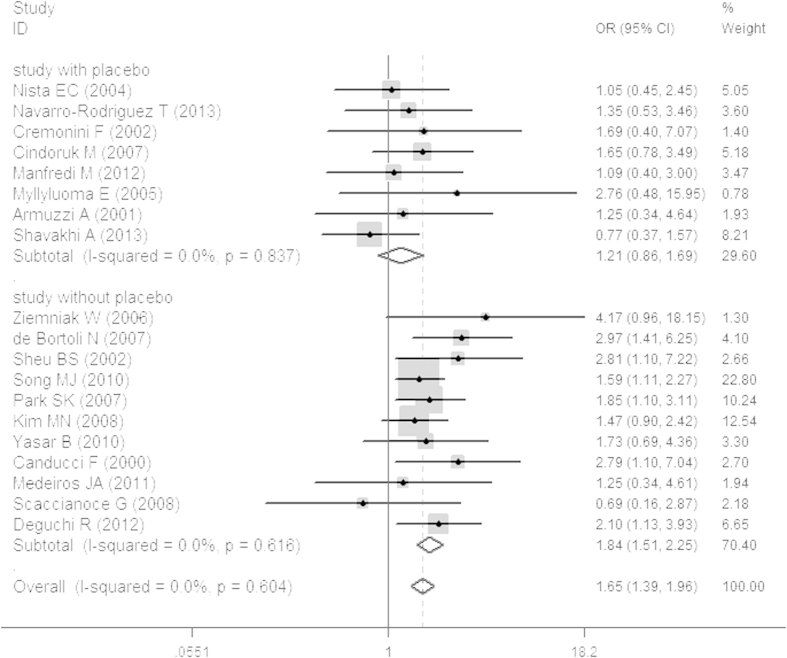
Meta-analysis of studies reporting on the eradication rate of *H. pylori* infections in the probiotics group *vs.* the placebo and non-placebo groups in ITT analysis and estimated the OR with a 95% confidence interval and weight percentage.

**Figure 3:**
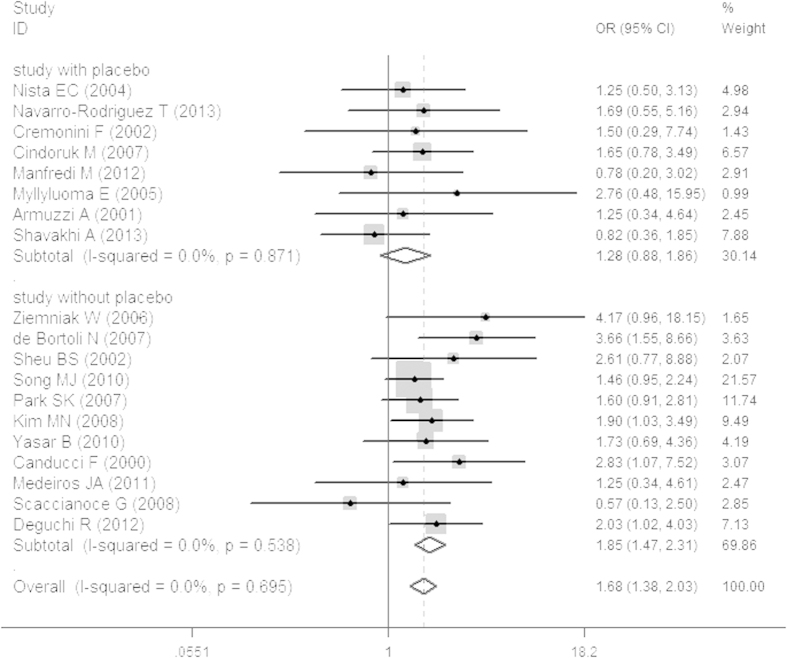
Meta-analysis of studies reporting on the eradication rate of *H. pylori* infection in the probiotics group *vs.* the placebo and non-placebo groups in PP analysis and estimated the OR with a 95% confidence interval and weight percentage.

**Figure 4:**
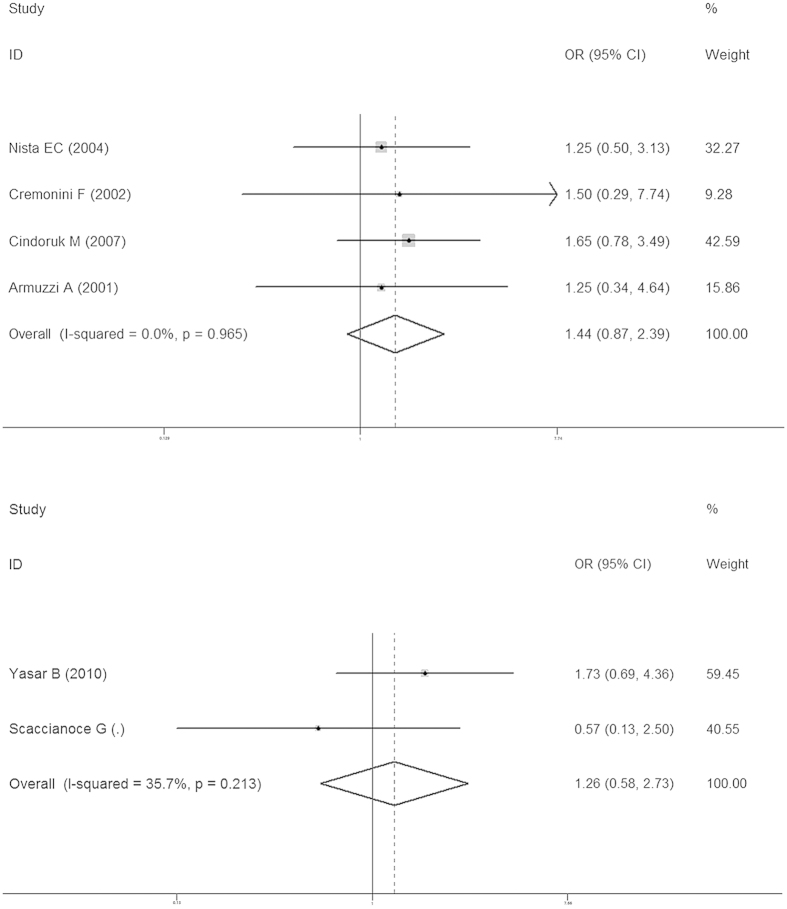
Meta-analysis of studies reporting on the eradication rate of *H. pylori* infection in the probiotics group *vs.* the placebo and non-placebo groups (probiotics with triple therapy plus a 14-day course of treatment) and estimated the OR with a 95% confidence interval and weight percentage.

PP results were used to represent the final eradication rates. Total eradication rates were 84.32 ± 10.66% and 77.87 ± 9.39% in the probiotics and control groups, respectively. In the studies with a placebo, the eradication rate was 84.07 ± 14.09% in the probiotics group and 79.22 ± 9.84% in the placebo group. In studies without a placebo, the eradication rate of was 84.48 ± 12.61% in the probiotics group and 77.04 ± 9.4% in the non-placebo group. In addition, our study revealed that in ITT analysis the probiotics group was 1.21 times more likely than the placebo group to achieve eradication of *H. pylori* infection (OR 1.21, 95% CI: 0.86, 1.69; Fig. 2) and 1.84 times more likely than the standard-therapy-alone group (OR 1.84, 95% CI: 1.51, 2.25; Fig. 2). In PP analysis, the probiotics group was 1.28 times more likely than the placebo group to achieve eradication of *H. pylori* infection (OR 1.28, 95% CI: 0.88, 1.86; Fig. 3) and 1.85 times more likely than the standard-therapy-alone group (OR 1.85, 95% CI: 1.47, 2.31; Fig. 3). Both ITT and PP analyses showed no statistically significant effect on eradication rates when the probiotics group was compared to the placebo group, but the probiotics group had a significantly higher eradication rate when compared to standard therapy alone. To avoid bias caused by the anti-*H. pylori* therapy scheme or the duration of probiotic use, we also conducted a sub-group analysis on treatment using probiotics with triple therapy plus a 14-day course of treatment. This showed that the probiotics group was not more likely to achieve the eradication of *H. pylori* infection (OR 1.44, 95% CI: 0.87, 2.39; Fig. 4) without statistical significance. In standard-therapy-alone groups, sub-group analysis on triple therapy plus a 14-day course of treatment also showed no statistical significance (OR 1.74, 95% CI: 0.96, 3.16; Fig. 4). Moreover, both ITT (*P* > *Z* = 0.108; *P* > *Z* = 0.436) and PP (*P* > *Z* = 0.108; *P* > Z = 0.640) meta-analyses had no publication bias under Begg’s funnel plot test.

In addition, we compared the eradication rates in placebo administration plus standard therapy with the standard therapy group in order to determine whether placebo treatment can improve eradication rates. Results revealed no statistical significance (79.22 ± 9.84% *vs.* 77.04 ± 9.4%; *P* = 0.816). However, the trend still showed a potentially higher eradication rate in the placebo plus standard therapy group. Thus, RCTs on placebo plus standard therapy *versus* standard therapy alone are needed to verify our hypothesis.

### Tolerance and adverse effects

The tolerance to the standard triple therapy itself may be affected by the probiotic supplementation. Among the included studies, only one clearly reported that there was no difference in tolerance between the probiotic and placebo groups (*P* = 0.833)^23^. Tolerance of standard therapy is affected by adverse effects. Therefore, we compared the adverse effects of diarrhea, nausea, vomiting, bloating, epigastric pain, constipation, headache and metallic taste. Between the probiotic group and the standard-therapy-alone group, we found that nausea (OR 0.43, 95% CI: 0.27, 0.7), vomiting (OR 0.3, 95% CI: 0.11, 0.86), diarrhea (OR 0.43, 95% CI: 0.21, 0.89), and constipation (OR 0.28, 95% CI: 0.13, 0.64) were improved in the probiotic group, whereas epigastric pain (OR 0.82, 95% CI: 0.48, 1.39), headache (OR 0.42, 95% CI: 0.11, 1.65), metallic taste (OR 0.69, 95% CI: 0.30, 1.58), and bloating (OR 0.54, 95% CI: 0.04, 6.64) were not different between the two groups (S1). Between the probiotic group and the placebo group, we found that nausea (OR 0.36, 95% CI: 0.21, 0.62), diarrhea (OR 0.33, 95% CI: 0.19, 0.57), and bloating (OR 0.5, 95% CI: 0. 3, 0.83) were improved in probiotic group, whereas epigastric pain (OR 0.58, 95% CI: 0.25, 1.32), vomiting (OR 0.71, 95% CI: 0.31, 1.62), and constipation (OR 0.56, 95% CI: 0.31, 1.01) were not different between the two groups (S2).

Nausea and diarrhea was clearly improved by probiotics, but it was not clear whether these two factors ultimately affected the curative effect.

## Discussion

This meta-analysis analyzed whether probiotic supplementation can improve the eradication rate of *H. pylori* infection based on standard therapy. In contrast to previously published meta-analyses^13^,^14^,^37^, we studied control groups given a placebo in order to determine whether placebo administration can influence eradication rates compared to probiotics. Studies without placebos were included for comparison. Our results revealed that the inclusion of probiotics to standard therapy does not increase eradication rates of *H. pylori* compared to a placebo.

Triple therapy for eradication of *H. pylori* infection is unsatisfactory throughout the world. *H. pylori* is the best known microbe that colonizes the gastric mucosa, causing gastric related diseases, as shown by Marshall^38^. However, Walker *et al*. revealed that the imbalance of other gastric microbiota can play an important role in affecting human health^39^. This may be an important factor in the lack of efficacy of standard therapy. In addition, the increasing resistance to antibiotics such as clarithromycin^6^, the frequency and duration of drug administration, and the occurrence of side effects can influence a patient’s compliance^40^.

Many studies, including the meta-analysis mentioned above, have reported that probiotic supplementation can safely improve eradication rates of *H. pylori* infection and decrease side effects, although some probiotic products have been shown to increase the risk of complications in a minority of specific patient groups^41^. Probiotics have been shown to be useful in several illnesses such as reducing the duration and severity of rotavirus gastroenteritis^42^, reducing the incidence of traveler’s diarrhea^43^, preventing and reducing relapses of *Clostridium difficile* colitis^44^, and anti-inflammation benefits for inflammatory bowel disease^45^. The mechanisms by which probiotics play their role have not been clearly defined. Many possible mechanisms have been put forward, such as inhibiting the adhesion of pathogenic bacteria to the intestinal wall and competing with microbial pathogens for a limited number of receptors present on the surface epithelium^46^, altering cytokine expression and the activity of intestinal-associated lymphoid tissue and epithelial cells^47^,^48^, and enhancing intestinal barrier function^49^. Therefore, it seems that probiotics can provide powerful supplements for the eradication of *H. pylori* infection. However, our findings were not sufficient to justify such expectations.

A placebo is a simulated or otherwise medically ineffectual treatment for a disease or other medical condition that intends to deceive the recipient. It is well-known that psychological phenomena are closely associated with gastric diseases^50^. In addition, the placebo effect generates alterations in the levels of systemic and enteric hormones^15^, and subject-expectancy effects^51^. The use of a placebo seems to play a potential role in treating *H. pylori* infection. Nevertheless, Asbjørn Hróbjartsson and Peter C. Götzsche indicated that there is little evidence for placebos having a strong clinical impact and that the formation of the placebo effect is a subjective factor^52^,^53^. In our study, merged data revealed non-significant results on eradication rates, which may be due to population selection bias of the groups included for Student’s *t*-test. Thus, more evidence is needed. RCTs including standard therapy plus placebo compared to standard therapy alone are needed in order to analyze whether placebo supplementation can improve the eradication rate of *H. pylori* infection.

This is the first meta-analysis and systemic review to compare probiotics plus standard therapy with placebo plus standard therapy for *H. pylori* infection. Although we reviewed many reports to strengthen our study, several limitations of this meta-analysis were inevitable. First, we lacked a large sample size and RCTs with sufficient case numbers in the placebo group. More large-sample RCTs would have increased the power of this analysis. Second, it is not clear whether differences in probiotics dose or composition, or the course of treatment, as well as differences in the specificity and accuracy of the diagnostic tools for *H. pylori* infection would influence the results. Third, the influence of adverse effects of probiotics should not be ignored, which may contribute to the eradication of *H. pylori* infections. In addition, due to lack of data, potentially relevant confounders such as race, smoking, lifestyle, and gene polymorphisms were not analyzed.

In conclusion, all the published research on probiotics plus standard therapy indicates that probiotics improve the eradication rate of *H. pylori* infection. However, in our study, we found that a 14-day triple therapy plus probiotics cannot improve eradication rates. In addition, the pooled data of our meta-analysis suggest that the use of probiotics plus standard therapy does not improve the eradication rate of *H. pylori* infection compared to placebo plus standard therapy, although probiotic supplementation can improve eradication rates compared to standard therapy alone. A placebo may achieve the same curative effect for the eradication of *H. pylori* infection compared to probiotics. Future research should pay more attention to the role of placebo in *H. pylori* eradication.

## Methods

### Search strategy and study selection

We searched studies published up to June 1, 2015, in PubMed, Ovid, EBSCO and Web of Science databases using the following terms: (Helicobacter pylori OR H. pylori OR Helicobacter infection OR Helicobacter* OR HP OR Helicobacter pylori (MeSH)), and (eradication OR treatment OR therapy OR disease eradication (MeSH)), and (probiotic OR probiotic* OR prebiotic OR yeast OR yogurt OR symbiotic OR Lactobacillus OR Bifidobacterium OR Saccharomyces OR Lactococcus OR Streptococcus OR Enterococcus OR probiotic(MeSH)). This study was limited to human and English-language randomized controlled trials (RCTs). In addition, the following criteria were used for selecting relevant studies: (1) study patients >18 years old; (2) study populations that have not been treated for *H. pylori* infection; (3) patients in the control group received standard therapy with or without a placebo; (4) patients in the experimental group received standard therapy with probiotics; (5) availability of relative information on *H. pylori* diagnosis and successful eradication rates; and (6) same administration of standard therapy for the experimental and control groups. Standard therapy was defined as triple treatment, sequential treatment, non-bismuth quadruple therapy, or bismuth-containing quadruple therapy^6^.

Combining the guideline^6^ and previous meta-analysis^13^, *H. pylori* infection diagnosed by at least one positive test result was considered confirmation of infection: (1) ^13^C/^14^C urea breath test (UBT); (2) rapid urease test (RUT); (3) *H. pylori* culture; (4) stool antigen test; or (5) histology of biopsy staining. The primary outcome of the study was the *H. pylori* eradication rate, which had to be confirmed by a negative ^13^C-UBT or other generally accepted method at least 4 weeks after the end of treatment. The secondary outcome measures were whether probiotics improve tolerance compared to the standard therapy. The adverse effects of interest were diarrhea, nausea, vomiting, bloating, epigastric pain, constipation, headache and metallic taste during anti-*H. pylori* therapy.

Eligibility of each study for inclusion was evaluated by two investigators. Any research-related disagreements were resolved by a third reviewer. The quality of RCTs included in this study was assessed using the Jadad scale^54^.

### Data abstraction

Two authors independently extracted data from all eligible studies, and a third author checked the results. Data were extracted into Microsoft Excel (2010 edition; Microsoft, Redmond, WA, USA) to effectively organize the data. The following data were obtained from included studies: base characteristics of patients, authors, year of publication, country of research, details of *H. pylori* eradication therapy, details related to interventions, primary outcomes, and diagnostic methods of *H. pylori* infection.

### Statistical Analysis

The ultimate goal of this study was to determine whether the probiotics group had a higher eradication rate than the placebo group. We also included groups without a placebo for comparison. Odds ratios (ORs) were used to measure the effect of probiotics plus standard therapy on *H. pylori* eradication rates in both intent-to-treat (ITT) and per protocol (PP). ORs were also used to measure the difference of adverse effects of interest between the probiotics group and the control group. Age and gender were analyzed by standardized mean difference (SMD) and OR, respectively. Statistical heterogeneity was analyzed with Chi-squared distribution, Chochran’s *Q*-test and *I*-squared statistics. A fixed-effects model (Mantel-Haenszel) was applied for meta-analysis if the *I*^*2*^ statistic was under 50% and/or the *Q*-test was not significant at *P*<0.05. We opted to stratify our analyses in this study with and without placebo. In addition, Begg’s funnel plot was used to assess publication bias. Data of eradication rates of standard therapy plus placebo and standard therapy alone were merged separately, and Student’s *t*-test analysis was conducted to compare these data. All analyses were carried out through the application of the commands metan and metabias in Stata 12.0 (Stata Corporation, Texas, USA), and Student’s *t*-tests were performed by SPSS 16.0 (IBM, Chicago, IL, USA). Associated data were calculated and plotted using GraphPad Prism 5 (Graph Pad, San Diego, CA, USA).

## Additional Information

**How to cite this article**: Lu, C. *et al*. Probiotic supplementation does not improve eradication rate of *Helicobacter pylori* infection compared to placebo based on standard therapy: a meta-analysis. *Sci. Rep.* **6**, 23522; doi: 10.1038/srep23522 (2016).

## Supplementary Material

Supplementary Information

## Figures and Tables

**Table 1:** Study characteristics with placebo.

Author/year	Country	Case number (probiotics/placebo)	Diagnostic Methods	Probiotics composition	Eradication Therapy	% Eradication in ITT (probiotics/placebo)	% Eradication in PP (probiotics/placebo)	Review of *H. pylori*
Nista *et al*.^22^	Italy	120 (60/60)	^13^C-UBT	*Bacillus clausii (B. clausii)*	(rabeprazole 20 mg bid + clarithromycin 500 mg bid + amoxicillin 1 g bid) × seven days + (probiotics or placebo) × 14 days	72.22/71.15	78/74	^13^C-UBT six weeks after the end of treatment.
Navarro- Rodriguez *et al*.^21^	Brazil	107 (55/52)	^13^C-UBT or histology	*Lactobacillus acidophilus, Lactobacillus rhamnosus, Bifidobacterium bifidum* and *Streptococcus faecium*	(lansoprazole 30 mg bid + tetracycline 500 mg bid + furazolidone 200 mg bid) × seven days + (probiotics or placebo) × 30 days	81.82/76.92	88.24/81.63	^13^C-UBT eight weeks after the end of treatment.
Cremonini *et al*.^18^	Italy	42 (21/21)	^13^C-UBT	*Lactobacillus GG* and *S. boulardii*	(rabeprazole 20 mg bid + clarithromycin 500 mg bid + tinidazole 500 mg bid) x seven days + (probiotics or placebo) × 14 days	81.82/72.73	85.71/80	^13^C-UBT 5–7 weeks after the end of treatment.
Cindoruk *et al*.^17^	Turkey	124 (62/62)	histology	*S. boulardii*	(lansoprazole 30 mg bid + Clarithromycin 500 mg bid + amoxicillin 1 g bid) × 14 days + (probiotics or placebo) × 14 days	70.97/59.68	70.97/59.68	^13^C-UBT six weeks after the end of treatment.
Manfredi *et al*.^19^	Italy	149 (73/76)	^13^C-UBT or SAT	*Lactobacilli* and *Bifidobacteria*	(esomeprazole 20 mg bid + amoxicillin 1 g bid) × first five days + (esomeprazole 20 mg bid + clarithromycin 500 mg bid + tinidazole 500 mg bid) × next five days + (probiotics or placebo) × 10 days (total)	89.04/88.16	92.86/94.37	SAT 8–10 weeks after the end of treatment.
Myllyluoma *et al*.^20^	Finland	47 (23/24)	^13^C-UBT	*Lactobacillus rhamnosus, L. rhamnosus, Bifidobacterium breve* and *Propionibacterium freudenreichii*)	(lansoprazole 30 mg bid + Clarithromycin 500 mg bid + amoxicillin 1 g bid) × seven days + (probiotics or placebo) × 28 days	91.30/79.17	91.30/79.17	^13^C-UBT four weeks after the end of treatment.
Armuzzi *et al*.^24^	Italy	60 (30/30)	^13^C-UBT	*Lactobacillus* GG	(rabeprazole 20 mg bid + Clarithromycin 500 mg bid + tinidazole 500 mg bid) × seven days + (probiotics or placebo) × 14 days	83.33/80	83.33/80	^13^C-UBT six weeks after the end of treatment.
Shavakhi *et al*.^23^	Iran	180 (90/90)	RUT or histology	*Lactobacillus* and *Bifidobacterium*	(omeprazole 20 mg bid + Clarithromycin 500 mg bid + amoxicillin 1 g bid + bismuth 240 mg bid) × 14 days + (probiotics or placebo) × 14 days	76.67/81.11	82.14/84.88	^13^C-UBT four weeks after the end of treatment.

**Table 2:** Study characteristics without placebo.

Author/year	Country	Case number (probiotics/control)	Diagnostic Methods	Probiotics composition	Eradication Therapy	% Eradication in ITT (probiotics/control)	% Eradication in PP (probiotics/control)	Review of *H. pylori*
Ziemniak *et al*.^36^	Poland	245 (53/192)	UBT	*Lactobacillus acidophilus*; *Lactobacillus rhamnosus*	(pantoprazole 40 mg bid + clarithromycin 500 mg bid + amoxicillin 1 g bid) × 10 days + (probiotics or not) × 10 days	96.23/85.94	96.23/85.94	UBT six weeks after the end of treatment.
de Bortoli *et al*.^26^	Italy	206 (105/101)	^13^C-UBT, SAT, RUT	*Lactobacillus plantarum*; *L. reuterii*; *Bifidobacterium infantis,* etc.	(esomeprazole 20 mg bid + clarithromycin 500 mg bid + amoxicillin 1 g bid) × seven days + (probiotics or not) × seven days	88.57/72.27	92.08/76.04	^13^C-UBT eight weeks after the end of treatment.
Sheu *et al*.^33^	China	160 (80/80)	Histology, RUT	*Lactobacillus-*; *Bifidobacterium-*	(lansoprazole 30 mg bid + clarithromycin 500 mg bid + amoxicillin 1 g bid) × seven days + (probiotics or not) × 28 days	91.25/78.75	94.81/87.5	^13^C-UBT eight weeks after the end of treatment.
Song *et al*.^34^	Korea	661 (330/331)	Histology, RUT	*S. boulardii*	(omeprazole 20 mg bid + clarithromycin 500 mg bid + amoxicillin 1 g bid) × seven days + (probiotics or not) × 28 days	80/71.6	85.44/80.07	^13^C-UBT four weeks after the end of treatment.
Park *et al*.^31^	Korea	352 (176/176)	Histology	*Bacillus subtilis*; *Streptococcus faecium*	(omeprazole 20 mg bid + clarithromycin 500 mg bid + amoxicillin 1 g bid) × seven days + (probiotics or not) × 56 days	83.52/73.3	85.47/78.66	^13^C-UBT four weeks after the end of treatment.
Kim *et al*.^29^	Korea	347 (168/179)	^13^C-UBT, histology, RUT	*L. acidophilus*; *L. casei*; *L. casei*; *S. thermophilus*	(PPI bid + clarithromycin 500 mg bid + amoxicillin 1 g bid) × seven days + (probiotics or not) × 21 days	79.17/72.07	87.5/78.66	^13^C-UBT four weeks after the end of treatment.
Yasar *et al*.^35^	Turkey	76 (38/38)	Histology	*Bifidobacterium*	(pantoprazole 40 mg bid + clarithromycin 500 mg bid + amoxicillin 1 g bid) × 14 days + (probiotics or not) × 14 days	65.79/52.63	65.79/52.63	^13^C-UBT four weeks after the end of treatment.
Canducci *et al*.^25^	Italy	120 (60/60)	^13^C-UBT, histology	*Lactobacillus acidophilus*	(Rabeprazole 20 mg bid + clarithromycin 250 mg tid + amoxicillin 500 mg tid) × seven days + (probiotics or not) × 10 days	86.67/70	88.14/72.41	^13^C-UBT four weeks after the end of treatment.
Armuzzi *et al*.^16^	Italy	120 (60/60)	^13^C-UBT	*Lactobacillus*	(pantoprazole 40 mg bid + clarithromycin 500 mg bid + tinidazole 500 mg bid) × seven days + (probiotics or not) × 14 days	80/76.6	80/80.7	^13^C-UBT six weeks after the end of treatment.
Medeiros *et al*.^30^	Portugal	62 (31/31)	Culture	*Lactobacillus acidophilus*	(esomeprazole 20 mg bid + clarithromycin 500 mg bid + amoxicillin 1 g bid) × eight days + (probiotics or not) × eight days	83.87/80.65	83.87/80.65	^13^C-UBT 6–7 weeks after the end of treatment.
Scaccianoce *et al*.^32^	Italy	31 (15/16)	Histology	*Lactobacillus plantarum*; *L. reuteri*; *Bifidobacterium Longum,* etc.	(lansoprazole 30 mg bid + clarithromycin 500 mg bid + amoxicillin 1 g bid) × seven days + (probiotics or not) × 14 days	53.33/62.5	53.33/66.67	^13^C-UBT 4–6 weeks after the end of treatment.
Deguchi *et al*.^27^	Japan	229 (115/114)	Culture, histology, RUT	*L. gasseri*	(rabeprazole 10 mg bid + clarithromycin 200 mg bid + amoxicillin 750 mg bid) × seven days + (probiotics or not) × 28 days	82.61/69.3	85.59/74.53	^13^C-UBT 8 weeks after the end of treatment.
Imase *et al*.^28^	Japan	14 (7/7)	Not mentioned	CBM588	(lansoprazole 30 mg bid + clarithromycin 400 mg bid + amoxicillin 750 mg bid) × seven days + (probiotics or not) × seven days	100/87	100/87	Not mentioned

**Table 3:** Basic characteristics of the included studies.

Author of study	Age (probiotics group)	Age (control group)*	M/F (probiotics group)	M/F (control group)*	Type of patients included
Nista *et al*.	46 ± 13	43 ± 13	33/27	22/38	Free of gastrointestinal symptoms
Navarro- Rodriguez *et al*.	50.4	48.4	21/34	19/33	51 PU patients and 56 dyspepsia patients
Cremonini *et al*.	–	–	–	–	Free of gastrointestinal symptoms
Cindoruk *et al*.	45.82 ± 13.35	47.56 ± 13.53	26/36	18/44	Dyspepsia patients
Manfredi *et al*.	46.4	50.6	39/34	37/39	Free of gastrointestinal symptoms
Shavakhi *et al*.	42.3 ± 13.3	42.2 ± 13.2	49/41	60/30	Patients with history of PU
Myllyluoma *et al*.	57.3	53.8	10/13	8/16	Free of gastrointestinal symptoms
Armuzzi *et al*.	–	–	–	–	Free of gastrointestinal symptoms
Ziemniak *et al*.	44.4 ± 13.3	43.7 ± 10.3	14/39	78/114	Patients with PU or gastritis
de Bortoli *et al*.	51.5±13.7	50.1 ± 15.2	56/49	54/47	Free or mild of gastrointestinal symptoms, among 25 PU patients
Sheu *et al*.	47.8	45.9	40/40	38/42	84 PU patients and 76 dyspepsia patients
Song *et al*.	49.76±11.7	49.84 ± 11.4	185/145	219/112	Patients with gastrointestinal symptoms, among 492 PU patients
Park *et al*.	45.2±19.8	47.6 ± 18.5	96/80	95/81	Patients with gastrointestinal symptoms, among 142 PU patients
Kim *et al*.	48.1 ± 12.4	53.7 ± 12.0	71/97	89/90	113 PU patients and 234 dyspepsia patients
Yasar *et al*.	38.32 ± 10.66	36.95 ± 8.62	11/27	14/24	Dyspepsia patients
Canducci *et al*.	–	–	–	–	Dyspepsia patients or patients with history of PU
Armuzzi *et al*.	–	–	–	–	Free of gastrointestinal symptoms
Medeiros *et al*.	50.7	53.8	18/13	14/17	Patients with history of PU
Scaccianoce *et al*.	50	48	7/8	6/10	Free of gastrointestinal symptoms
Deguchi *et al*.	55.9	57.8	76/39	68/46	Patients with history of PU
Imase *et al*.	–	–	–	–	Patients with history of PU

*In this table, the control group contains groups with or without a placebo.

**PU = peptic ulcer.
