# Supplementary material for: Probiotic supplementation does not improve eradication rate of Helicobacter pylori infection compared to placebo based on standard therapy: a meta-analysis
Source: Sci Rep. 2016 Mar 21;6:23522. doi: 10.1038/srep23522 (PMC4800733; doi:10.1038/srep23522)
Supplement: Supplementary Information [file srep23522-s1.pdf]

Probiotic supplementation does not improve eradication rate of Helicobacter pylori infection compared to placebo based on standard therapy: a meta-analysis

S1: Meta-analysis showed the distribution of diarrhea, nausea, vomiting, bloating, epigastric pain, constipation, headache and metallic taste in the probiotics group vs. non-placebo groups and estimated the OR with a 95% confidence interval and weight percentage.

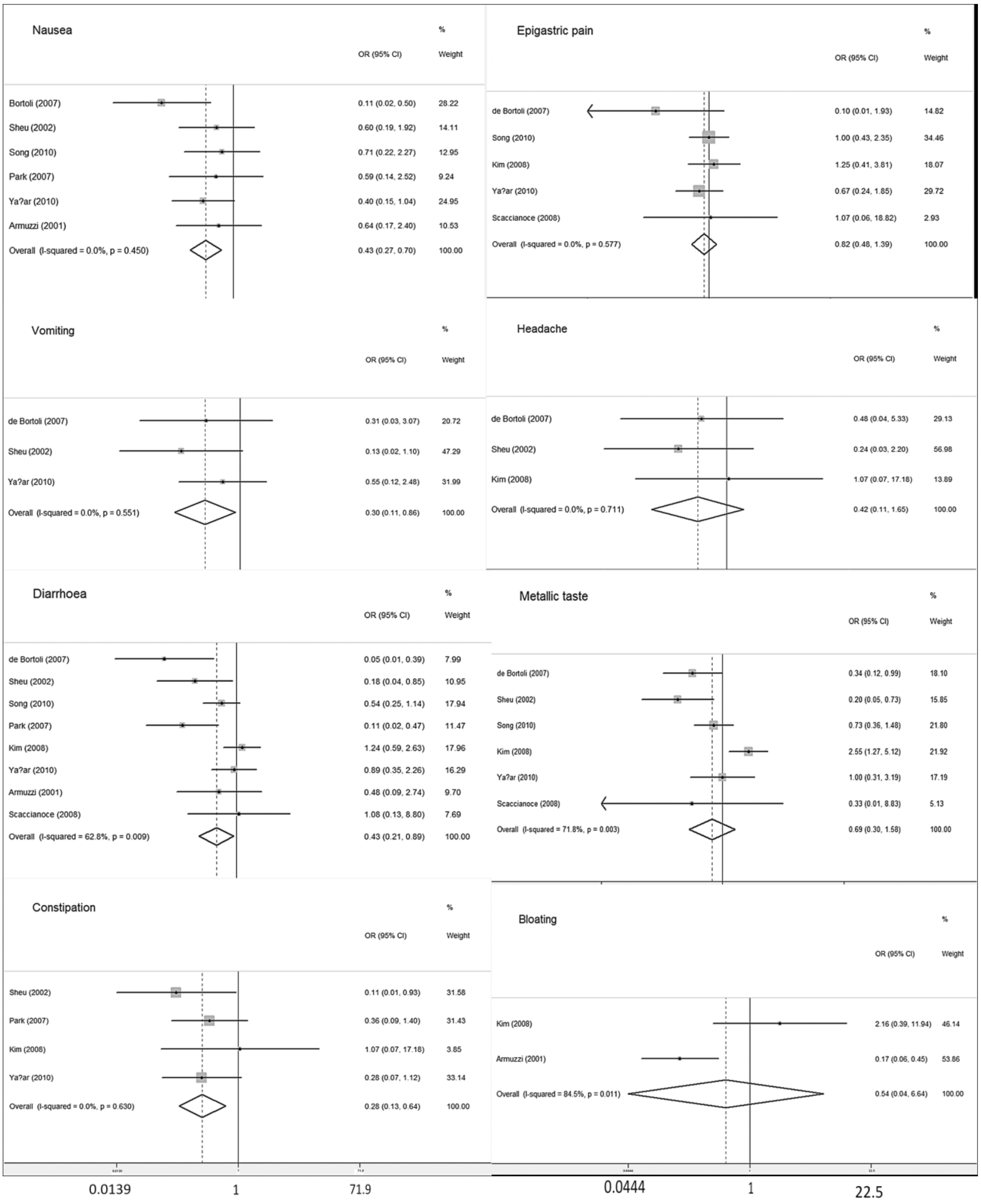

S2: Meta-analysis showed the distribution of diarrhea, nausea, vomiting, bloating, epigastric pain, constipation in the probiotics group vs. placebo groups and estimated the OR with a 95% confidence interval and weight percentage.

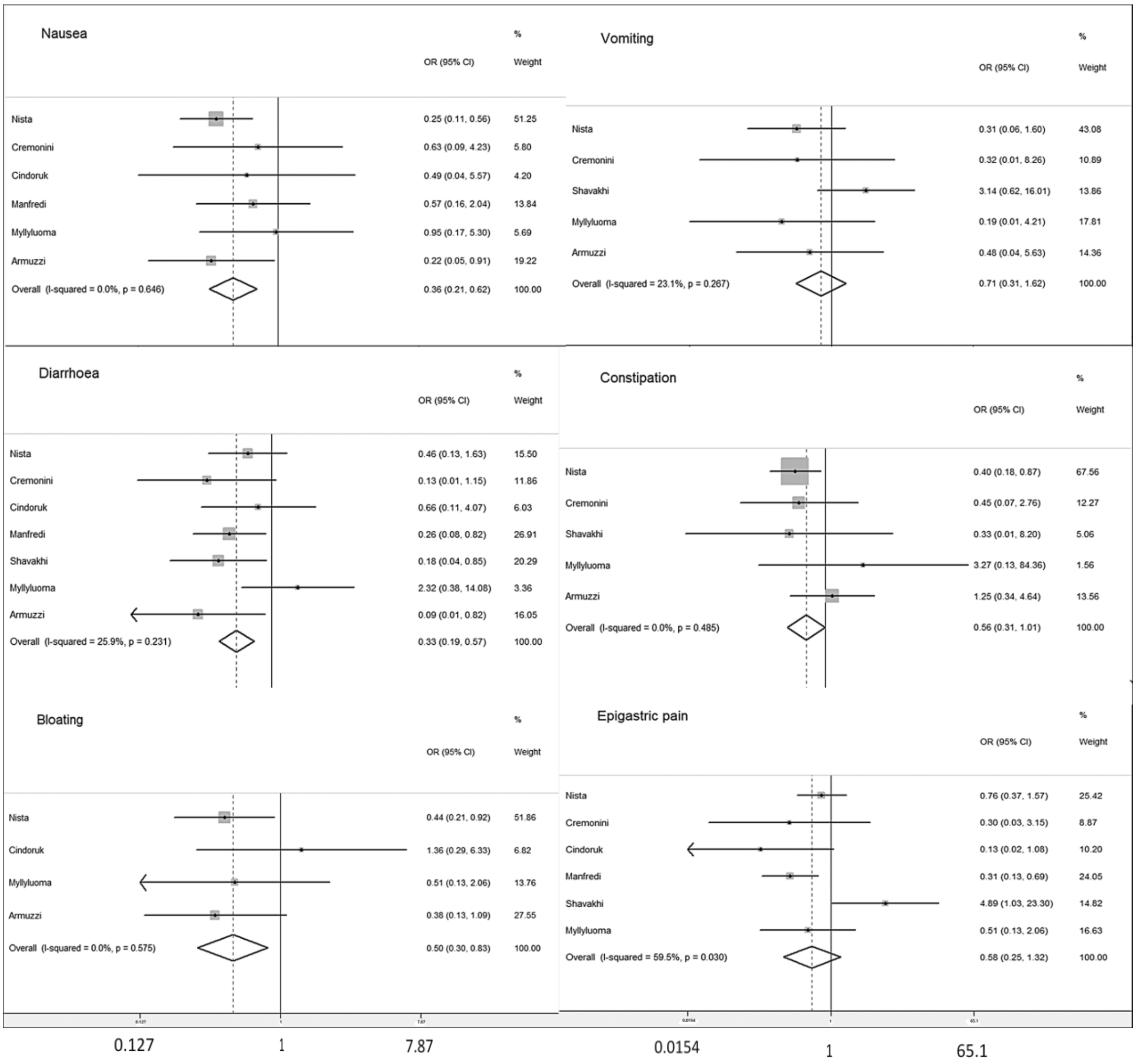

Nausea
OR (95% CI)
% Weight
Nista 0.25 (0.11, 0.56) 51.25
Cremonini 0.63 (0.09, 4.23) 5.80
Cindoruk 0.49 (0.04, 5.57) 4.20
Manfredi 0.57 (0.16, 2.04) 13.84
Myllyluoma 0.95 (0.17, 5.30) 5.69
Armuzzi 0.22 (0.05, 0.91) 19.22
Overall (I-squared = 0.0%, p = 0.646) 0.36 (0.21, 0.62) 100.00
Vomiting
OR (95% CI)
% Weight
Nista 0.31 (0.06, 1.60) 43.08
Cremonini 0.32 (0.01, 8.26) 10.89
Shavakhi 3.14 (0.62, 16.01) 13.86
Myllyluoma 0.19 (0.01, 4.21) 17.81
Armuzzi 0.48 (0.04, 5.63) 14.36
Overall (I-squared = 23.1%, p = 0.267) 0.71 (0.31, 1.62) 100.00
Diarrhoea
OR (95% CI)
% Weight
Nista 0.46 (0.13, 1.63) 15.50
Cremonini 0.13 (0.01, 1.15) 11.86
Cindoruk 0.66 (0.11, 4.07) 6.03
Manfredi 0.26 (0.08, 0.82) 26.91
Shavakhi 0.18 (0.04, 0.85) 20.29
Myllyluoma 2.32 (0.38, 14.08) 3.36
Armuzzi 0.09 (0.01, 0.82) 16.05
Overall (I-squared = 25.9%, p = 0.231) 0.33 (0.19, 0.57) 100.00
Constipation
OR (95% CI)
% Weight
Nista 0.40 (0.18, 0.87) 67.56
Cremonini 0.45 (0.07, 2.76) 12.27
Shavakhi 0.33 (0.01, 8.20) 5.06
Myllyluoma 3.27 (0.13, 84.36) 1.56
Armuzzi 1.25 (0.34, 4.64) 13.56
Overall (I-squared = 0.0%, p = 0.485) 0.56 (0.31, 1.01) 100.00
Bloating
OR (95% CI)
% Weight
Nista 0.44 (0.21, 0.92) 51.86
Cindoruk 1.36 (0.29, 6.33) 6.82
Myllyluoma 0.51 (0.13, 2.06) 13.76
Armuzzi 0.38 (0.13, 1.09) 27.55
Overall (I-squared = 0.0%, p = 0.575) 0.50 (0.30, 0.83) 100.00
Epigastric pain
OR (95% CI)
% Weight
Nista 0.76 (0.37, 1.57) 25.42
Cremonini 0.30 (0.03, 3.15) 8.87
Cindoruk 0.13 (0.02, 1.08) 10.20
Manfredi 0.31 (0.13, 0.69) 24.05
Shavakhi 4.89 (1.03, 23.30) 14.82
Myllyluoma 0.51 (0.13, 2.06) 16.63
Overall (I-squared = 59.5%, p = 0.030) 0.58 (0.25, 1.32) 100.00
0.127
1
7.87
0.0154
1
65.1
